# Root morphological responses to population density vary with soil conditions and growth stages: The complexity of density effects

**DOI:** 10.1002/ece3.7868

**Published:** 2021-07-07

**Authors:** Shu Wang, Lei Li, Dao‐Wei Zhou

**Affiliations:** ^1^ College of Forestry Forest Ecology Research Center Guizhou University Guiyang China; ^2^ Jiangxi Province Key Laboratory of Watershed Ecosystem Change and Biodiversity, Center for Watershed Ecology, Institute of Life Science and School of Life Sciences Nanchang University Nanchang China; ^3^ Northeast Institute of Geology and Agroecology Chinese Academy of Sciences Changchun China

**Keywords:** belowground interaction, growth stage, intraspecific competition, resource availability, root allocation

## Abstract

**Aim:**

How plants cope with increases in population density via root plasticity is not well documented, although abiotic environments and plant ontogeny may have important roles in determining root response to density. To investigate how plant root plasticity in response to density varies with soil conditions and growth stages, we conducted a field experiment with an annual herbaceous species (*Abutilon theophrasti*).

**Methods:**

Plants were grown at low, medium, and high densities (13.4, 36.0, and 121.0 plants m^−2^, respectively), under fertile and infertile soil conditions, and a series of root traits were measured after 30, 50, and 70 days.

**Results:**

Root allocation increased, decreased, or canalized in response to density, depending on soil conditions and stages of plant growth, indicating the complex effects of population density, including both competitive and facilitative effects.

**Main conclusions:**

Root allocation was promoted by neighbor roots at early stages and in abundant resource availability, due to low‐to‐moderate belowground interactions among smaller plants, leading to facilitation. As plants grew, competition intensified and infertile soil aggravated belowground competition, leading to decreased root allocation in response to density. Root growth may be more likely restricted horizontally rather than vertically by the presence of neighbor, suggesting a spatial orientation effect in their responses to density. We emphasized the importance of considering effects of abiotic conditions and plant growth stages in elucidating the complexity of density effects on root traits.

## Graphical abstract

The research revealed the complexity of root response to density, which may increase, decrease, or canalize, depending on the strength of above‐ and belowground interactions, which varied with soil conditions or growth stage.

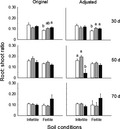

## 1 INTRODUCTION

Population density is a major biotic environmental factor in nature. For plants, increasing density can result in variations in multiple resources and intraspecific interactions (Casper & Jackson, 1997), influencing growth and responses. Therefore, increased density can have far more complicated influences on plants than merely effects on aboveground and/or belowground competition. In spite of much literature on plant responses to competition (Cahill, 2003; Murphy & Dudley, 2007), or shade/light signals due to increased density (Bongers et al., 2018; Forster et al., 2011), few empirical studies have focused on how plants respond to population density (Forster et al., 2011; Maliakal et al., 1999; Wang et al., 2017).

Without consideration of such complexity, studies on plant responses to competition also revealed contradictory conclusions, as follows: (a) Neither above‐ nor belowground conspecific competition alters root allocation (Cahill, 2003; Casper et al., 1998); (b) intraspecific interactions among plants enhance root:shoot ratio (Gersani et al., 2001; O'Brien et al., 2005); and (c) root mass ratio is lower at high versus low density (Forster et al., 2011; Poorter et al., 2012, 2016). Although these studies have contributed to our knowledge, they cannot substitute investigation on plant plasticity in response to density in an integrated view. Inconsistent results may be due to variations in density treatments, plant growth stage, and abiotic conditions. Additionally, biomass allocation or root:shoot ratio is also crucial and morphological root traits may also have important roles in facilitating plant adaptation to increased density.

Abiotic environmental factors may determine plant response to density in several ways (Forster et al., 2011). A deficiency of belowground resources can aggravate belowground competition (Casper & Jackson, 1997; Schenk, 2006), whereas increased soil resources can result in a shift in competition from occurring primarily belowground to primarily aboveground (Tilman, 1988; Wilson & Tilman, 1991), with increased interactions between above‐ and belowground competitions (Cahill, 1999). Consequently, soil conditions must affect plant response to density (Poorter et al., 2012), although that is poorly characterized (but see Weigelt et al., 2005). Above‐ and belowground competition elicited independent responses, and soil nutrients did not affect root response to the presence of neighbors (Murphy & Dudley, 2007). Perhaps, the low‐nutrient regime did not cause true nutrient deficiencies, especially when competition was not intense. Substantial abiotic effects may result from effects of low versus high levels of many resources or infertile versus fertile soil conditions.

Temporal heterogeneity of density effects is important. In a dense population, as plant size increases, competition intensity first increases and then weakens (Hutchings & Budd, 1981). Regarding allometric growth, a plant changes significantly in allocation pattern with various developmental phases (Harper & Ogden, 1970; Weiner, 2004). However, analyses of covariance or allometry based on a single stage cannot eliminate ontogenetic effects, as plasticity of allometric relationships in response to density varies with plant stages (Li et al., 2013). Therefore, density studies should include multiple growth stages.

Finally, based on meta‐analyses, laboratory‐grown plants experience different abiotic and biotic environments and much shorter growth periods, compared to those grown in fields, which may strongly affect plant morphology and physiology (Poorter et al., 2016). Therefore, studies should be done under field conditions (Gratani, 2014; Poorter et al., 2016), with the benefit of assessing root foraging traits such as main root length and lateral root length, in physically unrestricted spaces. Responses to density may differ in root mass allocation and morphological traits, as morphological traits often respond earlier than mass traits (Wang et al., 2017). Different root morphological traits may also vary in response to density, as neighbor roots can have different effects on main roots and lateral roots in terms of both space occupation and resource utilization.

Here, we conducted a field experiment by subjecting plants of an annual species *Abutilon theophrasti* to three densities, under fertile and infertile soil conditions, to measure a series of root morphological traits at three stages of plant growth, in order to investigate whether and how plant root response to density varies with soil conditions and plant growth stage. We tested the following hypotheses: (a) Higher density reduces root mass allocation, but has mixed effects on root morphological traits; (b) compared to fertile soil, infertile soil intensifies root responses to density; and (c) responses of root traits to density intensify and then weaken as plant growth continues.

## 2 MATERIALS AND METHODS

### 2.1 Studied species

*Abutilon theophrasti* Medicus (Malvaceae) is native to China and India but now spreads worldwide. It is an annual weedy species, erect with stout stems, growing to a height of 1–1.5 m. Through rapid growth, it can reach reproductive maturity within 90 days and complete its life cycle in ~5 months (McConnaughay & Coleman, 1999), with substantial plasticity in allocation, morphology, and architecture in response to varying environmental factors (McConnaughay & Bazzaz, 1992). It colonizes relatively nutrient‐rich habitats, being ubiquitous in open fields, on roadsides, and in gardens.

### 2.2 Experimental design

The experiment was conducted in 2007 at the Pasture Ecological Research Station of Northeast Normal University, Changling, Jilin Province, China (44°45′N, 123°45′E). Seeds were collected from local wild populations near the research station in late August 2006 and dry stored at −4°C. We used a split‐plot design, with soil conditions as the main factor and density and block as a subfactor. Two large plots were assigned as two (infertile and fertile) soil conditions; each was divided into nine 2 × 3 m subplots and randomly arranged with three treatments of densities and blocks. Seeds of *A*. *theophrasti* were sown on 7 June 2007, with interplanting distances of 30, 20, and 10 cm, to reach target plant densities of 13.4, 36, and 121 plants per m^−2^, assigned as low‐, medium‐, and high‐density treatments, respectively. Most seeds emerged 4 days after sowing. Seedlings were thinned to the target densities at the four‐leaf stage. Plots were hand‐weeded when necessary and watered regularly.

We established the infertile soil conditions as a plot using the original soil (aeolian sandy soil) of an experimental field at the station that had been used annually for many years. In contrast, fertile soil conditions were established by covering the other large plot with 5–10 cm virgin soil transported from a nearby meadow with no cultivation history (meadow soil), with contrasting nutrient contents of the two soil conditions (Wang et al., 2017). The meadow soil was close to the experiment field; the latter used to be meadow but had been reclaimed for experimental use since the research station was established. Therefore, the soil of the experimental field was the same type as the meadow soil, but with lower nutrient content. Covering the other plot with meadow soil led to a greater amount of soil or nutrients for the fertile soil treatment and also thicker soil layers of the fertile plot than the infertile one. To ensure consistency, the soil that was brought in was finely crushed and uniformly spread over the entire plot and compacted. Seeds were sown into all plots at the same burial depth and sowing rate.

### 2.3 Data collection and analysis

Plants were harvested at 30, 50, and 70 days of plant growth, representing developmental stages of early vegetative growth, late vegetative or early reproductive growth, and middle‐to‐late reproductive growth, respectively. At each stage, six individual plants were randomly chosen from each plot, making a total of 6 replicates ×3 plots ×3 densities ×2 soils ×3 stages = 324 samplings. For each individual plant, the following traits were measured if applicable: diameter at the basal of the main root, length, and number of lateral roots (≥1 mm in diameter along the main root). Morphological root traits were not measured at 30 days of growth due to small plant sizes. Each individual plant was then separated into roots, stems, petioles, leaves, reproductive organs, and branches (if any), enveloped, respectively, oven‐dried at 75°C for 2 days, and weighed.

All statistical analyses were conducted using SAS statistical software (SAS Institute 9.0 Inc., 2002). All traits were used including shoot mass, root mass, total biomass, and root:shoot ratio, and morphological traits of main root length, main root diameter, lateral root number, and lateral root length. To minimize variance heterogeneity, all data were log‐transformed before statistical analyses. Three‐way ANOVA and ANCOVA were used to evaluate overall effects of growth stage, soil conditions, and population density and their interactions on all traits, with total biomass nested in growth stage as a covariate in three‐way ANCOVA. Within each soil condition at each stage, effects of density were analyzed with one‐way ANOVAs for total mass and one‐way ANCOVAs for all the other traits, with total mass as a covariate. For a given trait, it was considered to exhibit apparent plasticity whenever plant size (total biomass) accounted for significant variation in its response to density, and any variation in its expression that was independent of total biomass was considered an indication of true plasticity (after removal of size effects) (McConnaughay & Coleman, 1999; Weiner, 2004). Multiple comparisons used the least significant difference (LSD) method of the general linear model (GLM) program in one‐way ANCOVA, which also produced adjusted mean values and standard errors.

## 3 RESULTS

Growth stage, soil condition, and population density had significant effects on total biomass and almost all other traits (Table 1). Interactions between stage and soil conditions, between stage and density, and between soil conditions and density were also significant for most mass traits, whereas interactions between stage and soil condition were also significant for most morphological traits.

**TABLE 1:** Three‐way ANOVAs on log‐transformed total biomass (TM) and ANCOVAs on log‐transformed root traits, with growth stage (GS), soil conditions (SC), and population density (PD) as effects, and log_10_(TM) as a covariate in ANCOVA

Trait	TM (*df* = 1)	GS (*df* = 2)	SC (*df* = 1)	PD (*df* = 2)	GS*SC (*df* = 2)	GS*PD (*df* = 4)	SC*PD (*df* = 2)	SC*GS*PD (*df* = 4)
TM		2,601.19***	121.88***	60.54***	42.40***	7.71***	7.15***	3.11*
SM	6,784.72***	133,581***	6,656.14***	2,906.51***	2.43	3.95**	3.72*	1.99
RM	4,938.17***	157.76***	102.18***	219.23***	3.36*	2.88*	3.01*	1.23
R/S	1.32	24.77***	7.05**	0.14	2.16	3.13*	3.50*	1.41
MRL	5.51**	54.37***	1.50	9.03***	16.14***	3.03*	0.81	1.44
MRD	131.22***	86.52***	374.02***	135.81***	11.91***	2.24	0.61	2.95
LRL	0.73	57.36***	19.03***	74.13***	61.99***	1.88	0.11	2.17
LRN	38.97***	7.04**	106.81***	42.70***	2.09	0.38	2.89	3.44*

Note

Degrees of freedom for the error terms were TM, SM, RM, and R/S 281 in three‐stage analyses; other traits 198 in two‐stage analyses.

Abbreviations: LRL, lateral root length; LRN, lateral root number; MRD, main root diameter; MRL, main root length; R/S, root:shoot ratio; RM, root mass; SM, shoot mass.

* *p* < .05

** *p* < .01

*** *p* < .001.

**FIGURE 1:**
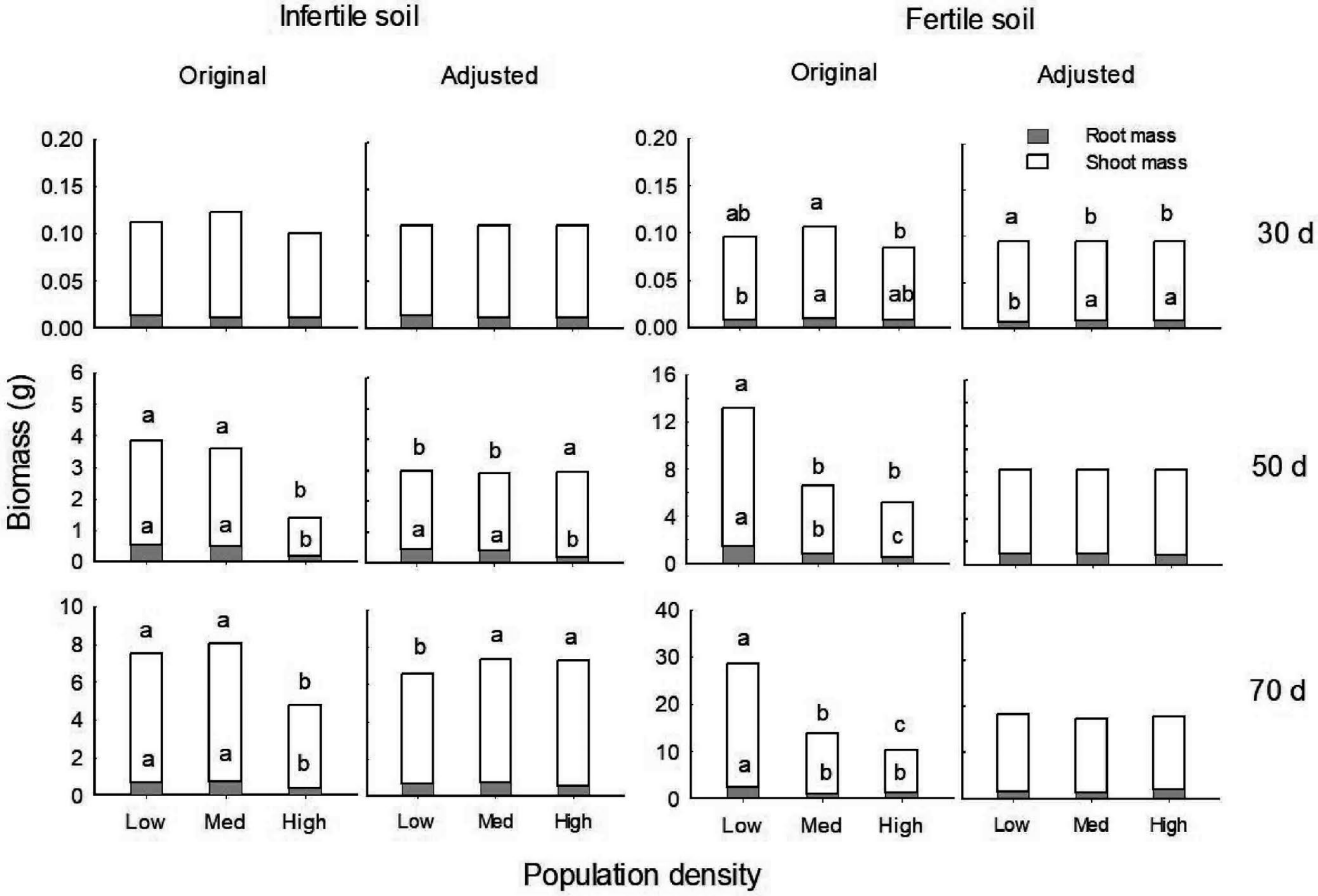
Original (left) and adjusted (right; by removing effects of total mass) mean values (±*SE*) of plant root mass (gray) and shoot mass (white) at low, medium, and high densities in infertile and fertile soil conditions at 30, 50, and 70 days of growth. Different letters denoted significant differences between density treatments (*p* < .05) within each soil condition and stage

**FIGURE 2:**
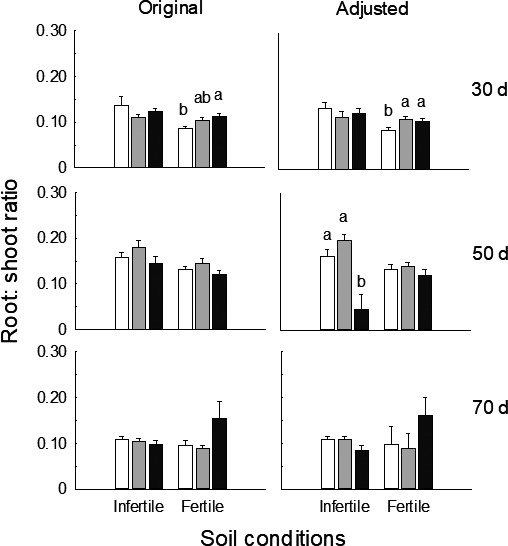
Original (left) and adjusted (right; by removing effects of total mass) mean values (±*SE*) of root:shoot ratio for individuals at low (white), medium (gray), and high (black) densities under infertile and fertile soil conditions at 30, 50, and 70 days of growth. Different letters denoted significant differences between density treatments (*p* < .05) within each soil condition and stage

**FIGURE 3:**
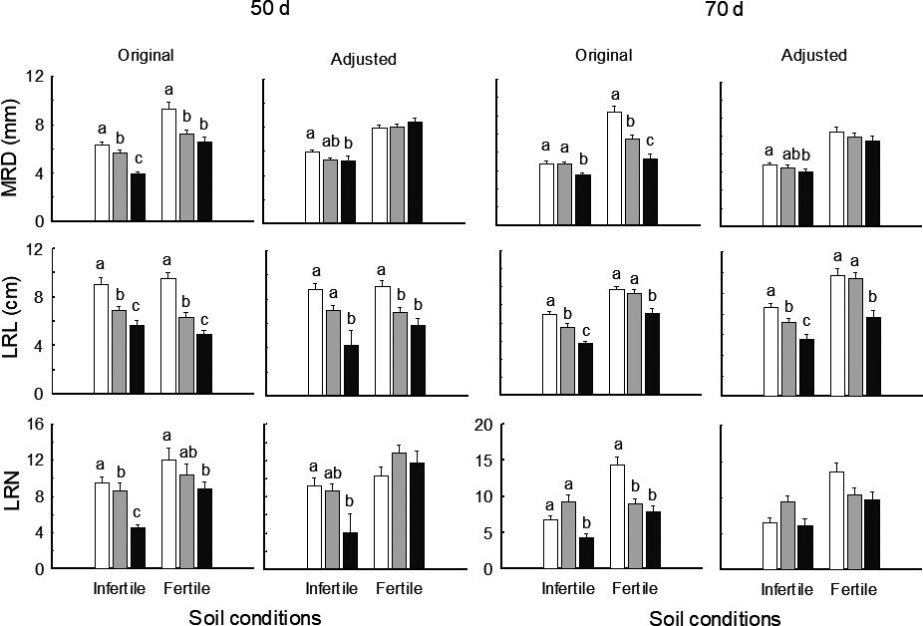
Original (left) and adjusted (right; by removing effects of total mass) mean values (±*SE*) of main root diameter (MRD), lateral root length (LRL), and number (LRN) at low (white), medium (gray), and high (black) densities under infertile and fertile soil conditions at 50 and 70 days of growth. Different letters denoted significant differences between density treatments (*p* < .05) within each soil condition and stage

Total biomass was decreased by infertile versus fertile soil and by the increase of density across all soil conditions and stages (*p* < .05; Table 1; Figure 1). Plant size (or total biomass) accounted for a significant amount of variation in all traits, except for root:shoot ratio and lateral root length (Table 1). After removal of size effect, effects of stage, soil, density, and stage and soil interactions were significant for most traits. Therefore, true plasticity in response to density occurred in all traits except main root length, and responses of these traits varied with soil conditions and/or growth stages (Table 2; Figures 2 and 3). For main root length, density effect was significant in three‐way ANCOVA, but not in one‐way ANCOVA, indicating apparent plasticity (Tables 1 and 2).

**TABLE 2:** *F*‐values for one‐way ANOVAs on log‐transformed total biomass (TM) and ANCOVAs on log‐transformed root traits, with population density (PD) as effect in two soil conditions at 30, 50, and 70 days of growth, and log_10_(TM) nested within growth stage as a covariate in ANCOVAs

Trait	Infertile soil			Fertile soil		
	*N*	TM (*df* = 1)	PD (*df* = 2)	*N*	TM (*df* = 1)	PD (*df* = 2)
30 days						
TM	37		1.69	46		2.64
SM	37	3,479.65***	1.57	46	13,194.4***	**3.62** *
RM	37	79.05***	1.34	46	39.57***	2.79
R/S	37	0.15	1.37	46	21.47***	2.87
50 days						
TM	49		**39.40** ***	53		**13.98** ***
SM	49	11,954.6***	**11.98** ***	53	22,725.7***	2.27
RM	49	96.34***	**7.45** **	53	451.34***	2.34
R/S	49	1.00	**7.98** **	53	0.06	2.35
MRL	49	19.24***	1.80	53	10.68**	3.26
MRD	49	269.48***	**4.74** *	53	447.95***	0.41
LRL	49	16.64***	**13.25** ***	53	56.20***	**8.57** ***
LRN	42	21.44***	**8.53** ***	53	52.16***	1.48
70 days						
TM	51		**9.17** ***	46		**19.83** ***
SM	51	1,515.25***	**3.31** *	46	3,697.80***	2.38
RM	51	176.49***	1.89	46	87.67***	1.53
R/S	51	0.00	1.49	46	0.46	1.59
MRL	51	0.01	2.83	46	1.19	0.46
MRD	51	19.89***	**4.16** *	46	161.14***	1.64
LRL	51	18.39***	**15.45** ***	46	0.98	**11.52** **
LRN	51	34.58***	**6.23** **	46	45.90***	0.80

Note

*N* indicates the number of individual values in each soil and stage combination.

Abbreviations: LRL, lateral root length; LRN, lateral root number; MRD, main root diameter; MRL, main root length; R/S, root:shoot ratio; RM, root mass; SM, shoot mass.

* *p* < .05

** *p* < .01

*** *p* < .001.

At 30 days of growth, neither soil condition nor population density affected total biomass (Table 2; Figure 1), whereas medium and high versus low densities decreased shoot mass, increased root mass, and root:shoot ratio of plants in fertile soil (*p* < .05) but not for those in infertile soil (Figures 1 and 2). At 50 days and 70 days, more traits responded to density in infertile versus fertile soil (Table 2). In infertile soil, compared to low density, high density enhanced shoot mass, reduced root mass, and root:shoot ratio (*p* < .01) and lateral root number (*p* = .002) at 50 days, whereas medium and high densities enhanced shoot mass at 70 days (*p* < .05); however, there was no response to density in these traits in fertile soil at either stage (Figures 1, 2, 3). For both stages, lateral root length decreased with higher densities in both soil conditions (*p* < .01), whereas main root diameter was decreased by high density in infertile soil only (*p* < .05; Figure 3).

## 4 DISCUSSION

Root mass allocation increased, decreased, or canalized with increased density, depending on soil conditions and growth stages, whereas root morphological traits generally decreased with increased density. Therefore, we inferred that plant root responses to increasing density were complex and not simple, including multiple responsive trends within and among traits. Increasing density may lead to above‐ and/or belowground interactions among plants; the overall results can be either competition or facilitation. In this study, plant total mass was generally decreased by high and medium densities, indicating competitive effects dominated over facilitative effects. Meanwhile, variations in plasticity of root traits due to soil conditions and growth stages also revealed plant strategies in dealing with biotic stresses.

### 4.1 Variations in plasticity of root allocation

For the first stage, plants were not large and branchy enough to interfere with each other above ground; therefore, most interactions were belowground. In that regard, belowground competition may occur prior to and be of a larger magnitude than aboveground competition (Weigelt et al., 2005). In response to density, there was no plasticity in total mass at this stage, but there were variations in plasticity of root allocation with soil conditions, including (a) increased root:shoot ratio with higher densities in fertile soil and (b) canalized root:shoot ratio in infertile soil (Table 3a). The greater root mass at medium versus low density indicated an effect of belowground facilitation; however, no response in total mass suggested the belowground interaction was not intense. When plants in a dense population are not large enough, they are more likely to promote root proliferation of each other via belowground interactions at low‐to‐moderate levels, since small plants may have little chance to shade each other or compete for belowground resources.

**TABLE 3:** Experimental results and associated information of the present study (in bold font) and other similar studies, showing responses of mass traits to (a) belowground interaction only; (b) aboveground interaction only; and (c) both above‐ and belowground interactions or population density

Shoot mass	Root mass	Root:shoot ratio	Total mass	Interaction intensity	Growth space	Density treatment	Soil conditions	Growth stage	Species	Source
(a) Belowground interaction only										
**NS** *^a^*	**NS**	**NS**	**NS**	**L**	**Plot**	**13, 36, 121 m^−2^**	**Infertile**	**30 days**	***Abutilon theophrasti***	**Present study**
−	+	+	**NS**	**L**↑	**Plot**	**13, 36, 121 m^−2^**	**Fertile**	**30 days**	***Abutilon theophrasti***	**Present study**
UA	UA	+	+	M	2.06 L	1, 4 pot^−1^	UA	42 days	*Glycine max*	Murphy and Dudley (2007)
NS	+	+	+	M	500 ml	1, 2 pot^−1^	UA	60 days	*Pisum sativum*	O'Brien et al. (2005)
NS	+	+	+	M	13.27 L	1, 2 pot^−1^	UA	110 days	*Glycine max*	Gersani et al. (2001)
UA	UA	+	−	H	500 ml	1, 4, 8, 16 pot^−1^	UA	−	*Avena sativa*	Li et al. (2016)
UA	UA	NS	−	H↓	Plot	1 m^−2^	UA	120 days	*Achillea millefolium L*. et al.	Cahill (2003)
(b) Aboveground interaction only (light quantity and quality treatments)										
UA	UA	NS	NS	L‐M	516 ml	0.5, 1.9 R: FR	UA	42 days	*Glycine max*	Murphy and Dudley (2007)
UA	UA	−	NS	L‐M↑	115 ml	0.2, 1.0 R: FR; 65%, full PAR	UA	80–170 days	*Acacia implexa*	Forster et al. (2011)
UA	UA	−	−	H	3 L	25, 50, 75%shade	UA	1–2 years	*Picea sitchensis* et al.	Kennedy et al. (2007)
UA	UA	NS	−	H↓	3.5 L	7, 100 m^−2^	UA	60 days	*Abutilon theophrasti*	Casper et al. (1998)
(c) Both above‐ and belowground interactions (density)										
+	−	−	−	**H**	**Plot**	**13, 36, 121 m^−2^**	**Infertile**	**50 days**	***Abutilon theophrasti***	**Present study**
UA	UA	−	−	H	Plot	80, 1,076 m^−2^	−	60 days	*Impatiens capensis*	Maliakal et al. (1999)
**NS**	**NS**	**NS**	−	**H↓**	**Plot**	**13, 36, 121 m^−2^**	**Infertile**	**70 days**	***Abutilon theophrasti***	**Present study**
**NS**	**NS**	**NS**	−	**H↓**	**Plot**	**13, 36, 121 m^−2^**	**Fertile**	**50, 70 days**	***Abutilon theophrasti***	**Present study**
UA	UA	NS	−	H↓	115 ml	1, 15, 50 pot^−1^	−	80–170 days	*Acacia implexa*	Forster et al. (2011)

Note

The intensity of interactions was estimated as low (L), moderate (M), and high (H) levels according to the responses of traits. NS, +, and − indicate no significant effects, an increase, and decrease, respectively, in response to interactions, and UA indicates unavailable information.

Plant–plant interactions can produce either competitive or facilitative results (Callaway, 2007; Callaway & Walker, 1997), depending on plant growth stages or stress levels (Callaway, 1995; Callaway & Pennings, 2000; Callaway & Walker, 1997). Abiotic conditions may shift the balance of competitive and facilitative effects (Armas et al., 2011; Bertness & Callaway, 1994) and thereby the overall outcome of plant–plant interactions (Michalet et al., 2011). Under more stressful conditions, facilitative effects may be more common, and competitive effects may be attenuated (Dohn et al., 2013; Gómez‐Aparicio et al., 2004; Lortie & Callaway, 2006). However, Foxx and Fort (2019) also reported stronger root competition and total competition at low versus high water availability. Inconsistent results indicated the intensity of interactions, and the overall result of competition and facilitation was more likely to depend on plant size rather than on abiotic conditions. Low‐to‐moderate levels of interactions are more likely to produce facilitative effects (Casper & Jackson, 1997), especially in the absence of aboveground interactions. In this study, plants were not large enough to compete with neighbors above ground at 30 days, and the belowground interaction was moderate enough to produce facilitative effects. Regardless, the intensity of plant–plant interactions should be determined by plant growth stage and resource levels in combination. Plants in infertile soil of this study may have been too small to have any interactions with each other, whereas fertile soil may have promoted root growth, leading to moderate belowground interactions and thus facilitation. It is noteworthy that all factors that affect plant size, such as pot space, number of neighbors (or growing density), and sizes of species (due to age or genetic nature), can also influence the intensity of plant–plant interactions (Table 3).

At the second stage, interaction intensity continued to increase as plants grew larger; when both above‐ and belowground interactions occurred, total mass was decreased by intense interaction under both soil conditions (Table 3c). For this stage, root:shoot ratio was either (a) decreased by increased density in infertile soil, consistent with Li et al. (2016), or (b) kept stable in fertile soil, consistent with Cahill (2003). Increased density either did not affect root allocation (Forster et al., 2011) or decreased root allocation (Maliakal et al., 1999), depending on resource levels. Similar decreases in root:shoot ratio by intraspecific interaction also occurred in dry soil, compared to no response to intraspecific interaction in wet soil (Wang & Callaway, 2021). When plants grew large enough to compete for resources, competitive effects counteracted facilitative effects, leading to canalized root allocation in fertile soil. Meanwhile, as belowground resources decreased, competition among plants transformed from primarily aboveground to primarily belowground (Grime, 1973, 1979). Low resource availability of infertile soil may have intensified belowground competition (Cahill, 1999; Casper & Jackson, 1997; Schenk, 2006; Tilman, 1988; Wilson & Tilman, 1991), leading to decreased root allocation.

At the third stage, no response to density in root allocation in infertile soil suggested that the intensity of interactions among plants first increased and then decreased over time (Hutchings & Budd, 1981; Wang et al., 2017), due to elimination of small plants. Aboveground interactions may occur only as plants grew large enough, when belowground interactions also intensified. In these studies, root mass allocation decreased or canalized in response to reduced R:FR (red: far red) and/or shading, or aboveground interaction only, depending on the strength of competition (Table 3b).

### 4.2 Comparing plasticity of various root traits

Various root morphological traits differed in response to density: Root traits mainly expand horizontally, such as lateral root length and number and main root diameter, were more likely to decline with increased density. However, those expanding into greater soil depths, such as main root length, were less affected by density. This implied an effect of spatial orientation in response to density in root propagation. In that regard, plants restrict expansion horizontally rather than vertically in the presence of neighbor (Gundel et al., 2014) or avoid neighbors in the horizontal direction (Zhang et al., 2020). For example, high density increased roots of apple (*Malus* sp.) into deeper rather than upper soil layers (Atkinson et al., 1976), similar to other results (Mason et al., 1982; Pearson & Jacobs, 1985). Belowground modules of bulb that enlarged horizontally were suppressed by high density, whereas roots that grew into greater depths did not (Li et al., 2011). The presence of neighbors in the horizontal direction may have reduced nutrient available, making it inefficient to forage a greater range for satisfactory resources (Semchenko et al., 2007; Zhang et al., 2020). Therefore, as long as the deeper space is available, it is more profitable to expand roots vertically to acquire resources of greater depths, rather than scrambling for deficient upper soil resources (Gundel et al., 2014). Such spatial orientation effect of density in this study was also supported by the alleviated reduction in lateral root traits due to increased density in fertile versus infertile soil.

## 5 CONCLUSIONS

Our results showed root mass allocation increased, decreased, or canalized, in response to the increase of density, depending on the strength of interactions that varied with soil conditions and growth stages, indicating the complexity of density effects. Root allocation was promoted by increased density at early growth stages and under high resource availability, as a result of low‐to‐moderate belowground interactions and its facilitative effects due to appropriate plant sizes. As plants grew larger over time, the intensity of competition increased and may have counteracted facilitative effects, leading to canalized root allocation in response to density in abundant resources, or decreased root allocation by increased density due to aggravated belowground competition in deficiency of resources. Various root morphological traits can exhibit contrasting responses to density, as a result of spatial orientation effect in their responses to density. We emphasized the importance of considering effects of abiotic conditions and plant growth stages in elucidating the complexity of density effects on root traits.

## CONFLICT OF INTEREST

The authors have no conflict of interest to declare.

## AUTHOR CONTRIBUTIONS

**Shu Wang:** Conceptualization (lead); Data curation (lead); Formal analysis (lead); Funding acquisition (lead); Investigation (lead); Methodology (lead); Project administration (lead); Resources (lead); Software (lead); Supervision (lead); Validation (lead); Visualization (lead); Writing‐original draft (lead); Writing‐review & editing (lead). **Lei Li:** Project administration (supporting); Writing‐review & editing (supporting). **Dao‐Wei Zhou:** Conceptualization (supporting); Funding acquisition (supporting); Methodology (lead); Supervision (supporting).

## Data Availability

Data are available in the Dryad Digital Repository: http://doi.org/10.5061/dryad.ngf1vhhs2.
